# Who Is Writing About Women in STEM in Higher Education in the United States? A Citation Analysis of Gendered Authorship

**DOI:** 10.3389/fpsyg.2019.02979

**Published:** 2020-01-21

**Authors:** Heidi Blackburn, Jason Heppler

**Affiliations:** ^1^Research and Instruction Services, Dr. C.C. and Mabel L. Criss Library, University of Nebraska Omaha, Omaha, NE, United States; ^2^Archives and Special Collections, Dr. C.C. and Mabel L. Criss Library, University of Nebraska Omaha, Omaha, NE, United States

**Keywords:** authorship, science, technology, engineering, mathematics, gender, higher education

## Abstract

The purpose of this study was to identify trends in the representation of female authorship regarding the topic of the status of women in science, technology, engineering, and mathematics (STEM) in higher education in the United States from 2007 to 2018 in prominent interdisciplinary journals. We conducted a comprehensive search for articles and collected the genders of the first and senior authors from 647 citations. We assessed the number of male versus female authors, the percentages of female first authors and senior authors, and the percentage of female corresponding authors for each year. Additionally, we also analyzed the citations to determine the journals and publishers who produced the most literature in this area. Women constituted 59% overall authorship and 34% first authorship. The top publishers in this area include Sage Publications, Springer/Nature, and Elsevier. Women constituted 60% of the first authors in STEM literature on the status of women and 38% of senior authors. Although there was growth over time in first authorship in STEM literature written by women, they continue to remain a minority in senior authorship. We suggest it is women that are leading this discussion, highlighting the additional lift that women in STEM in higher education must make; researching and publishing on their own experiences as part of their self-advocacy.

## Introduction

In recent years, the emphasis on science, technology, engineering, and mathematics (STEM) education in the United States has had a tremendous impact on the professional literature generated on the topic. A major subset of this research focuses on the experiences of United States. women students and faculty, including recruitment and retention (Blackburn, 2017). These studies explore everything from student motivation (Graziano et al., 2012; Smith et al., 2012; Chumbley et al., 2015; Talley and Ortiz, 2017; Leaper and Starr, 2018), self-concept (Sax et al., 2015; Koul et al., 2016; Morton and Parsons, 2018), self-efficacy (Dugan et al., 2013; Verdín and Godwin, 2018), and identity (Robnett et al., 2015; Beals, 2016) to biases (Handley et al., 2015; LaCosse et al., 2016; Moss-Racusin et al., 2018), stereotypes (Cheryan et al., 2015; Barth et al., 2017; Banchefsky and Park, 2018), campus culture (Crenshaw et al., 2017; Dresden et al., 2018), and lived experiences (Maltese and Tai, 2011; Alexander and Hermann, 2016; Smith and Gayles, 2018). Studies covering women faculty members include barriers to tenure (Soto, 2014; Williams and Ceci, 2015; Skewes et al., 2017), promotion (Gumpertz et al., 2017), work-life balance (O’Brien and Hebl, 2008; Adamo, 2013; Pedersen and Minnotte, 2017), and administrative advancement (Avallone et al., 2013; Lopez et al., 2018).

Traditionally, women have had to, and continue to, champion for resources, access, benefits, and promotion when it comes to gender equity issues in the academic workplace (Feeney and Bernal, 2010; Bachman, 2011; Villablanca et al., 2011; Adamo, 2013; Beddoes and Pawley, 2014; Kachchaf et al., 2015; Su and Bozeman, 2016). Changes to policies and procedures are often achieved through service and committee work which are traditionally unequally shouldered by women faculty compared to men (Guarino and Borden, 2017). Women have to form their own committees and voice their concerns over equity disparities in addition to their official faculty duties (Bird et al., 2004; Misra et al., 2011) and while often doing “the lion’s share” of housework (Schiebinger and Gilmartin, 2010). Across all academic disciplines, women work to increase gender parity in the classroom, in the research lab, and on the tenure track. Women faculty in STEM disciplines must challenge gendered teaching loads (Carrigan et al., 2011), biased tenure and promotion practices (Soto, 2014), work-family imbalances (Bachman, 2011; Beddoes and Pawley, 2014; Myers, 2015; Tanenbaum, 2015), research (Cozzens, 2008; Howe et al., 2014; Deemer, 2015; Hart, 2016), harmful departmental policies (Holmes et al., 2016), and biased diversity hiring practices (Easley, 2013; King, 2013; Smith et al., 2015; Williams and Ceci, 2015). Women in STEM from underrepresented minorities (Armstrong and Jovanovic, 2017; Leggett-Robinson and Campbell Villa, 2019) and those on the LGBTQIA (Lesbian, Gay, Bisexual, Trans, Queer, Intersex, and Asexual) spectrum (Billimoria and Stewart, 2009; Patridge et al., 2014) have faced additional intersectional barriers, including institutional racism, tokenism, homophobia, and bullying (Armstrong and Jovanovic, 2015; Cascio, 2017).

Productivity, including first authorship and senior authorship, is another one of the spaces that women in research and academic publishing have faced barriers (Beddoes and Pawley, 2014; Aiston and Jung, 2015; Fishman et al., 2017; Bendels et al., 2018). In most STEM disciplines, author conventions dictate that the first author listed is usually an early career researcher, or project lead, while the last authors are generally senior researchers (Early et al., 2018; Holman et al., 2018). Customarily, the first author has contributed the most work and the sequence of subsequent authors are determined by the contributions of their work (Riesenberg and Lundberg, 1990; Tscharntke et al., 2007). However, Fox and Paine (2019) found journal submissions with female first authors obtained, on average, slightly worse peer−review scores and were more likely to be rejected after peer review in science journals.

The STEM disciplines continue to retain low numbers of women to these fields. Gómez Cama et al. (2016) analyzed 14 higher education journals and found that only 74 of the 6,459 articles published from 2000 to 2013 analyzed gender differences. The recruitment of women to STEM fields has historically been a difficult battle with “pipeline” methods (Espinosa, 2011; Cannady et al., 2014; Hurlock, 2014; Teo, 2014; Vazquez-Akim, 2014; Doerschuk et al., 2016; Makarova et al., 2016; Redmond-Sanogo et al., 2016; Bergeron and Gordon, 2017) and “pathways” methods (Heilbronner, 2009; Wang and Degol, 2013, 2017; Ashford et al., 2016; Clark et al., 2016; Heyman, 2016; Perez-Felkneri et al., 2017) being introduced with limited success. According to the National Science Foundation [NSF] and National Center for Science and Engineering Statistics [NCSES] (2019), the most recent statistics show that women have received half of the degrees at each level (bachelors, graduate, and doctoral) in the biological sciences but earned fewer than half of the degrees in the physical sciences. Likewise, women are leaving academic STEM programs, both as students (George-Jackson, 2011; Geisinger and Raman, 2013; Kahn and Ginther, 2015; Riegle-Crumb et al., 2016) and faculty (Xu, 2008; Burnett et al., 2012), highlighting conversations about retention and attrition (Diekman et al., 2010; Rask, 2010; Singh et al., 2013; Shedlosky-Shoemaker and Fautch, 2015; Xu, 2017).

Science, technology, engineering, and mathematics professional organizations insist on the importance of conversations surrounding the topic of the recruitment and retention of women because of a predicted need for a stronger future workforce (Cohoon, 2001; Avallone et al., 2013; Cech and Blair-Loy, 2019) but it was unclear who is leading this discussion and where it is taking place within the published literature due to the intersectionality of the topic. Does the responsibility to lead this conversation on recruitment and retention lie with the professional disciplines themselves or with the women they are recruiting? The assignment of who must lead this conversation tells those in the field whether the discussion about equity is a priority or not. If the responsibility falls to women authors alone, particularly in one-off publications, there may be an imbalance that should be rectified. We wanted to understand if there was a difference between women and men publishing on this topic as well as where they were choosing to publish this work to frame the conversation.

We addressed two research questions in this study:

(1) Which journals lead in publishing literature regarding women in STEM in higher education in the United States?
(2) Who is conducting research regarding women in STEM in higher education in the United States?
  (a) Are there more women or men researchers authoring articles regarding women in STEM in higher education in the United States?
  (b) Are there more women listed as first or senior authors in articles regarding women in STEM in higher education in the United States?
  (c) How frequently do authors write together in author networks in articles regarding women in STEM in higher education in the United States?
  (d) Where do authors publish research regarding women in STEM in higher education in the United States most frequently?

Specifically, this study extends the current frameworks of gender and authorship by exploring first and senior authorship status in articles published on the subtopic of women in STEM in both 2-year and university settings in the United States.

## Materials and Methods

For this data analysis, a convenience sample was taken and all the articles collected and analyzed were available through commercial database systems (including Google Scholar, Web of Knowledge, Scopus, ScienceDirect, ProQuest, and EBSCO) and came from peer-reviewed, scholarly journals and conference proceedings regarding studies conducted in the United States published from 2007 to 2018. This period was enough to provide reliable data to present trends. We used both journals and conference proceedings but excluded books, dissertations, and theses from this analysis as well as non-research pieces such as book reviews and editorials. Articles and conference proceedings were included based on their coverage of the topic of women in STEM in higher education, regardless of the original discipline of the publication, such as the sciences or social sciences.

We imported one thousand citations into a Zotero library and then exported them into a Microsoft Excel spreadsheet, using a customized output style. Once in Excel, we de-duped the results and identified off-topic articles through manual review of the authors, title, date, and journal fields. Many off-topic articles were incidental retrievals due to the occurrence of the word stem as related to stem cell or plant stem and we used 647 citations in the analysis. We standardized the titles as we entered the citation information into the database by referring to Ulrich’s Global Serials Directory. We sorted the results alphabetically by the titles of the journals in which the articles appeared. The total number of journals was determined, as well as the number of articles published by each source journal as well as publishing house. We sorted the journal list with the journal publishing the most articles listed at the top, followed by the journal that published the second most, continuing down the list.

After collecting the bibliographic information, we could proceed with visualizing the information. Using the R statistical language and the tidyverse library, we proceeded with data clean up that removed extraneous characters, null values, and split author names into first and last name columns. A second transformation of the data took our semicolon-delimited lists of authors and separated authors into their own rows while maintaining the appropriate bibliographic metadata. The resulting tidy data with an author now arranged on a separate row (Wickham, 2014) allowed us to move forward with the analysis. The code and resulting data can be found on GitHub.

All articles were affiliated with American higher education institutions. To determine authorship, we designated the gender of the first and last authors based on names listed within each article. If a single author published the article, we considered that author to be the first author. People identify on a broad spectrum of gender identities, including male, female, non-binary, and more. We recognize that gender identity is not visible but is an internal sense of one’s own gender. However, for the purposes of this study, author gender was categorized as male or female based on authors’ names using the knowledge that many names are associated with one gender or another (i.e., Rachel for women and José for men). We made a first pass on determining gender using the gender R package from rOpenSci (Mullen, 2018), and then confirmed those results and reviewed inconclusive results. If we could not ascertain the gender of the author from inspection of names, we performed a search for the author’s website with images based on traditional presentation of male or female or for articles recognizing the author’s preferred pronouns to determine the gender or as stated in the text by the author. If the gender was still uncertain after an exhaustive Internet search (we found no profile pages or images within the first 30 search listings as to how the author presents themselves), we designated the author’s gender as undetermined.

From the overall list of articles collected, we generated four subsets of data for analysis to help us address our questions. First, we separated out conference proceedings from journals so we could analyze the data without skewing the results toward proceedings. That left us with two sets of data: one for proceedings, and one for journal publications. With these subsets we could address our first and fourth questions by examine the frequencies of female and male authors as well as determining those journals most frequently publishing on women in STEM in higher education in the United States. Second, we sought to determine the order of authorship to explore whether female or male authors appeared as first author. This transformation included generating a numbered list that corresponded with each author’s contribution; the number assigned to the author reflected their order in authorship for any article contributions they provided. This subset allowed us to address our second question on first and senior authors. Any solo authors or first authors were assigned a “1,” while remaining authors were assigned a number based on the number of remaining authors on an individual article. From there, we could loop through each individual article and find the highest number for that article, which told us the senior authors on a given piece. To help answer our third question, we generated co-authorship network data and bi-model network data to explore (1) how frequently authors write with one another, and (2) which journals they publish in most frequently. From this dataset we could examine which authors wrote together and see which publications had the most frequent authors.

## Results

We divided the results into four categories: (1) publications in which articles about women in STEM in higher education were written, (2) differences in publication rates for men and women authors, (3) placement of women authors in first or senior (last) position, and (4) author networks between publications.

### Major Publications

Overall, just 3% percent of the publications accounted for 25% percent of the citations. Most of the articles were concentrated over the top ten publications (22%) and the rest were distributed within 286 journals (74%) and nine conference proceedings (4%). Only three of the top ten journals publishing articles on topics regarding women in STEM in higher education fell within the science disciplines, as defined by Ulrich’s Global Serials Directory Subject Classifications. Seven of the ten titles fell within social science disciplines, including the subcategories of psychology and education (see Table 1). The top journals were *Journal of Women and Minorities in Science and Engineering* (*n* = 21), *Sex Roles* (*n* = 21), and *Social Sciences* (*n* = 16).

**TABLE 1:** Journals publishing articles on women in STEM in higher education.

		Number of
Rank	Journal title	publications
1	Journal of Women and Minorities in Science and Engineering	21
2	Sex Roles	21
3	Social Sciences	16
4	Frontiers in Psychology	14
5	Journal of Science Education and Technology	13
6	Psychology of Women Quarterly	13
7	Research in Higher Education	13
8	Journal of Vocational Behavior	10
9	PLOS ONE	10
10	Journal of Diversity in Higher Education	8

In contrast, the top nine conference proceedings publishing articles on topics regarding women in STEM in higher education fell within the STEM disciplines, as defined by Ulrich’s Global Serials Directory Subject Classifications (see Table 2). The *American Society of Engineering Education (ASEE) Annual Conference and Exposition, Conference Proceedings* (*n* = 34) had the most publications and there was a steep decline in proceeding articles with *2007 37th Annual Frontiers in Education Conference* (*n* = 2) trailing in second. For overall publications, *American Society of Engineering Education (ASEE) Annual Conference and Exposition, Conference Proceedings* had the most publications (*n* = 34), followed by the *Journal of Women and Minorities in Science and Engineering* (*n* = 21), and *Sex Roles* (*n* = 21).

**TABLE 2:** Conference proceedings publishing articles on women in STEM in higher education.

		Number of
Rank	Conference proceeding title	publications
1	ASEE Annual Conference and Exposition, Conference Proceedings	34
2	2007 37th Annual Frontiers in Education Conference	2
3	2014 Institute of Electrical and Electronics Engineers (IEEE) Frontiers in Education Conference (FIE) Proceedings	1
4	7th Institute of Electrical and Electronics Engineers (IEEE) Gulf Cooperation Council (GCC) Conference and Exhibition	1
5	Association for Computing Machinery (ACM) Conference on Innovation and Technology in Computer Science Education	1
6	Association for Computing Machinery (ACM) International Conference on Measurement and Modeling of Computer Systems	1
7	American Institute of Physics (AIP) Conference Proceedings	1
8	Proceedings - 2016 International Conference on Computational Science and Computational Intelligence	1
9	Proceedings of the 7th Institute of Electrical and Electronics Engineers (IEEE) Integrated STEM Education Conference	1

From 2007 to 2018, the number of total articles about women in STEM in higher education in the United States. that were published by all authors across all publishers increased by 2,420%. On average, there was a 41% increase in published articles each year, with the largest increase between 2009 (*n* = 10) and 2010 (*n* = 24), resulting in an increase of 140%. Sage Publications (11.9%), Springer/Nature (11.75%), and Elsevier (9.89%) were the top publishers of articles (see Table 3). The top publications from Sage Publications included *Psychology of Women Quarterly, Journal of Career Assessment*, and *Personality and Social Psychology Bulletin*. Springer/Nature’s top publications included *Sex Roles, Journal of Science Education and Technology*, and *Research in Higher Education*, and Elsevier’s top publications included *Journal of Vocational Behavior, Economics of Education Review*, and *Computers & Education*.

**TABLE 3:** Top publishers of articles on women in STEM in higher education.

		Percentage of
Rank	Publisher	journals owned
1	Sage Publications	11.90
2	Springer/Nature	11.75
3	Elsevier	9.89
4	Taylor & Francis – Routledge	7.88
5	American Society of Engineering Education	5.41
6	John Wiley & Sons	4.17
7	American Psychological Association	3.86
8	Begell House	3.25
9	Frontiers	3.09
10	MDPI – AG	2.63

### Women and Men Authors

Through this analysis, we identified 1,967 unique authors that resulted in three categories: women (*n* = 1,173), men (*n* = 473), and unidentified authors (*n* = 353). Female authors comprised most of the authors (59.7%) and male authors (22.2%) and unidentified authors (17.9%) were almost equally split. From 2007 to 2018, the number of authors writing about women in STEM in higher education in the United States. across all publishers increased by over 2,252%. On average, there was a 43% increase in authors publishing on this topic each year.

### Women as First and Senior Authors

**FIGURE 1:**
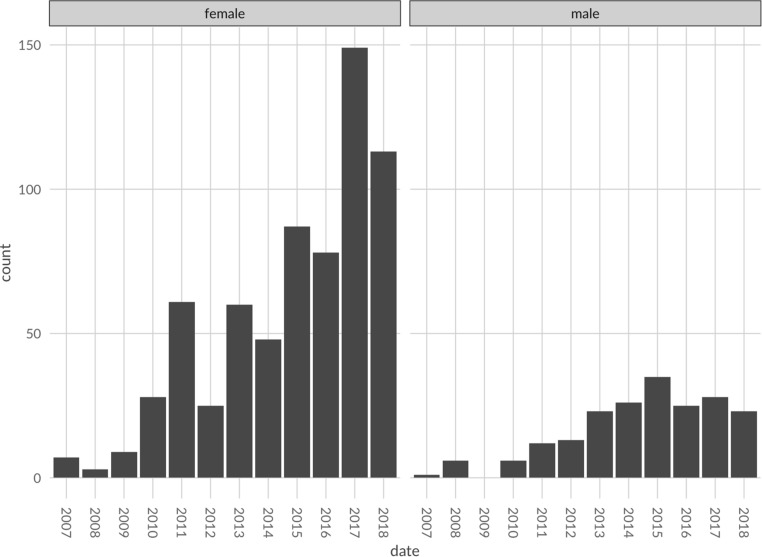
The count of first author’s gender by year.

The number of first, or solo, authors among all first authors were split, with 60.72% identified as women, 18% men, and 21.27% were undetermined. Women made up 38.5% of senior authors (author positions between 2 and 33) and men were 14.5% of senior authors (see Figure 1). Out of all unique first authors, 357 of them were women, 110 were men, and 88 were undetermined. On average, the percentage of female first authors grew by 39% between 2007 and 2018, while the percentage of male first authors grew by 13%.

### Author Networks and Publications

**FIGURE 2:**
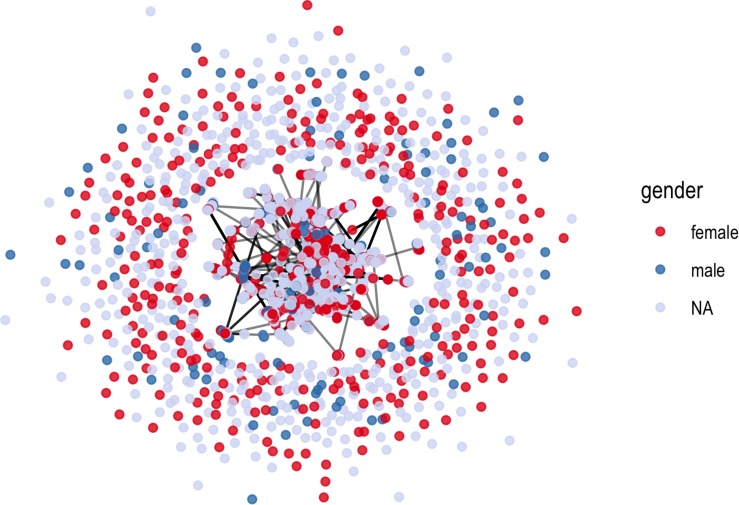
Co-authorship network with node color indicating an author’s gender.

The top ten authors published 3.24% of the works within the sample but authors also cluster and publish in a variety of science-specific and social science-specific journals. We found that 522 of the articles were authored by two or more authors (89%), with 125 solo-authored pieces. Within the co-authorship network, there were 1,536 distinct authors. Female authors who co-author accounted for 51% of authorship while male authors whom co-author accounted for 20.5% of the articles. Female first authors who also co-authored accounted for 20.1% of the articles while 39.5% of the female co-authors were listed as the senior author. No evidence was found of publishing networks between publication theme and the formation of strong author groups. While there are infrequent collaborations, authors mainly continue to author independently with little evidence of larger intra-network publishing community (see Figure 2). Furthermore, we can use a measure of graph density to look at all of the potential connections between nodes in the network, which measures a value between 0 and 1. The closer to 1 in density, the higher the number of connections in the graph. Despite the high number of co-authored pieces, the network density is 0.003 indicating that of all the possible links between nodes in the network there are very few that connect. What the visualization and the density measure tell us overall is that while co-authors may author together frequently, there is little evidence of broader collaboration among various author groups.

We also looked at the publishers within this sample to explore how they supported co-authored publications writing about women in STEM in higher education. There were ten journals that had the highest number of publications written by two or more authors (see Table 4). The top three publications that supported collaborative publications (ranked by percentage of collaborative articles published divided by total articles by that journal within the sample) were *Journal of Diversity in Higher Education* (100%), *Journal of Vocational Behavior* (100%), and *Social Sciences* (93%).

**TABLE 4:** Top journals with collaborative articles on women in STEM in higher education.

		Collaborative articles/
		total articles in
Rank	Journal/Publisher	sample (%)
1	Journal of Diversity in Higher Education/American Psychological Association	100
2	Journal of Vocational Behavior/Elsevier	100
3	Social Sciences/MDPI – AG	93.7
4	Frontiers in Psychology/Frontiers	92.8
5	PLOS ONE/PLOS	92.8
6	Journal of Women and Minorities in Science and Engineering/Begell House	90.4
7	Sex Roles/Springer/Nature	90.4
8	Psychology of Women Quarterly/Sage Publications	84.6
9	Journal of Science Education and Technology/Springer/Nature	69.2
10	Research in Higher Education/AABRI	61.5

## Discussion

The goal of the present study was to identify trends in the representation of female authorship regarding the topic of the status of women in STEM in higher education in the United States from 2007 to 2018 in prominent interdisciplinary journals. For this purpose, we reviewed 647 articles to review the publication and gender distribution of first authorship and senior authorship in 295 major journals and nine conference proceedings over the past decade. The top two publications, *American Society of Engineering Education (ASEE) Annual Conference and Exposition, Conference Proceedings* (5.26%) and *Journal of Women and Minorities in Science and Engineering* (3.25%) are well-known STEM publications. However, *Sex Roles* also accounted for 3.25% of the articles and is defined by Ulrich’s Global Serials Directory as a social science journal. In fact, 70% of the top ten journals fall within social sciences disciplines. Most of the publishers who are leading the conversation about women in STEM in higher education are found in the social sciences, not the science, technology, engineering, or mathematics disciplines. While nearly all the conference proceedings we found fell in the STEM domain, the journals in which authors publish most frequently on this topic are concentrated in education and psychology fields. Within the top social science journals in this sample, 63% of authors were women and 25% were first author.

By visualizing the collaborative nature of faculty productivity, we can more easily express some of the intangible social impacts of their work (Lewis and Alpi, 2017). In a comprehensive review of the JSTOR literature, West et al. (2013) showed that women historically have been underrepresented in the first author position and that women were underrepresented in the last author position. Lundine et al. (2018, p. 1754) note “Being the author of a paper, acting as a peer reviewer, and obtaining an appointment as an editorial board member, associate editor, or editor-in-chief are important recognitions for merit and promotion.” However, Beaudry and Larivière (2016) found that academic women publish less and receive fewer citations in health, natural sciences, and engineering journals and individuals with a higher proportion of female authors were ultimately cited less frequently. Because of these known inequities, there have been calls to increase women’s contributions to help combat the unconscious bias that persists in the scientific community by fundamentally shifting how female researchers are viewed and valued (Kaatz et al., 2014; Filardo et al., 2016). We observed that, overall, women constituted 60% of the authorship of studies on women in STEM in higher education, 60% of first authors, and 38.5% of senior authors in this sample. The first author (or lead author) of an article is commonly the person performing and directing the study, therefore, the gender of the first author may be an indicator of active involvement of women in researching this area. The last author of an article is often the person responsible for the study, and this status may be an indicator of the progression of women into more senior positions. Both positions continue to be coveted positions for those in academia, especially those who are establishing careers (Venkatraman, 2010). The number of women publishing on this topic is growing on average 30% faster each year than the number of men publishing. They are twice as likely to write collaborative papers compared to men but, overall, most of the articles continue to be independently authored papers.

The increase in female authors in STEM fields found in this study supports recent studies. Filardo et al. (2016) examined the representation of women as first authors in high impact general medical journals and found the number was significantly higher in 2014 than in 1994, but it has plateaued in recent years and had even declined in some journals. They suggest that the underrepresentation of research by women in these journals is still an important concern. Likewise, an analysis by West et al. (2013) revealed that there had been important gains in gender parity in first authorship across the natural sciences, social sciences, and humanities with the proportion of women first authors being even slightly higher than the overall proportion of female authorships. However, they also revealed that women in the last author position and proportion authoring overall continued to be disproportionately low. Holman et al. (2018, p. e2004956) confirmed this 5 years later stating women were “…substantially underrepresented as the last named author in the author list and as single authors and overrepresented as first authors relative to the overall authors gender ratio.” We concur by finding female authorship has increased over time but the percentage of women in in senior author position remains low. Holman et al. (2018, p. e2004956) suggest the gender gap is likely to “persist for generations, particularly in surgery, computer science, physics, and maths. The gap is especially large in authorship positions associated with seniority, and prestigious journals have fewer women authors.” Thus, with the results of the present study we extend the literature by confirming the findings of Liang et al. (2015), Long et al. (2015), and Larivière et al. (2013) regarding gender imbalances in female authorship.

### Limitations

Our study had several limitations. First, we based gender designation on name recognition through the data visualization process and looking up author pages. Therefore, we cannot be certain of the gender of authors. Second, we had a high percentage of authors of undetermined gender (17.9%). Unknown names were often names that were not consistent with common Western gender associations or represented authors whose first names were presented only as initials. Therefore, this can potentially lead to less reliable data in some of the journals analyzed. We performed a thorough Internet search of authors’ names, but if still unsure, we put them in a separate category. Third, one of the study’s limitations is the use of convenience sampling of the journals. Finally, the sample is restricted to research articles and conference proceedings whereas a future study may employ a larger sample, including non-research articles such as reviews, editorials, web/bibliographies, and opinions.

### Future Directions

Overall, 81.76% of the studies were written by women about the experiences of women in STEM in higher education. These findings lead us to posit several questions for future research, particularly regarding the engagement of men in the discussion as first and senior authors. Further discussion is needed for identifying reasons men are not conducting more peer-reviewed research on women in STEM in higher education when there were 23.5% more men than women employed full time in science and engineering in 2017 (National Science Foundation [NSF] and National Center for Science and Engineering Statistics [NCSES], 2019).

We would like to see the following questions in this area addressed in future studies:

• If 70% of the top publications are social science journals but a majority of science faculty review and cite journals in their own fields (Currie and Monroe-Gulick, 2013), how is this discussion being dispersed to the wider STEM professions? Does this hinder the conversation about recruiting and retaining women in STEM if researchers are missing important findings?
• What does it mean professionally for the STEM fields to have more women writing about women than men when they are in the numerical minority?
• Are there social or professional norms that prevent men from feeling comfortable engaging women on this topic? Do they feel it is not “their place” to explore or engage with this research because of the intersectionality of the issues women face?
• How and why do women feel they should author this research? Does their self-advocacy and advocacy for other women in STEM play a part in this decision?

Ruder et al. (2018) highlights the norms and practices of the “boys club” that persist in STEM in higher education institutions which privilege men while disadvantaging women. Tenured women faculty are more likely to do unrecognized emotional labor, which includes managing relationships, being the “face” of the department for recruiting efforts, and mentoring students. Most of the current studies about women in STEM in higher education focus on similar activities and this could be one reason why many of the authors are women. Men may be more protective of their research time, focusing on grants and publishing in other areas of research (Ruder et al., 2018). Likewise, this research may be seen as “institutional housekeeping” as defined by Bird et al. (2004, p. 195) because it represents “…the invisible and supportive labor of women to improve women’s situation within the institution.” This highlights the additional lift that women in STEM in higher education must make; researching and publishing on their own experiences as part of their self-advocacy.

## Conclusion

Female authors are leading the discussion by pursuing and publishing research concerning the recruitment and retention of women students, faculty, and staff in STEM. When a group of people feel seen and their voices are heard, they will feel a sense of community and inclusiveness, leading to higher retention in the fields. Most publications that are leading the conversation about women in STEM in higher education are found in the social sciences, not the science, technology, engineering, or mathematics disciplines. This leads the authors to believe that not only are women in STEM facing barriers in the classroom and the lab, the publications they are likely reading for professional development and research are not publishing articles about their experiences. While nearly all the conference proceedings we found fell in the STEM domain, the journals in which authors publish most frequently on this topic are concentrated in education and psychology fields. The need for equity work for among genders publishing in STEM disciplines appears to be far from over.

## Data Availability Statement

The datasets for this study can be found in the GitHub at doi: 10.5281/zenodo.3228477.

## Author Contributions

Both authors contributed to the conception, design of the study, manuscript revision, read, and approved the submitted version. HB collected the data, organized the database, and wrote the first draft of the manuscript. JH performed the statistical analysis and data visualization manipulations, and wrote sections of the manuscript. In relation to the context of this study on female authors, HB is the senior author on this work due to the nuances of the library science profession placing emphasis on the first author position.

## Conflict of Interest

The authors declare that the research was conducted in the absence of any commercial or financial relationships that could be construed as a potential conflict of interest.
